# Vibration and Trajectory Tracking Control of Engineering Mechanical Arm Based on Neural Network

**DOI:** 10.1155/2022/4461546

**Published:** 2022-07-22

**Authors:** Xinjun Lei, Yunxin Wu

**Affiliations:** ^1^State Key Laboratory of High Performance Complex Manufacturing, Central South University, Changsha 410083, China; ^2^SANY Automobile Manufacturing Co.,Ltd, Changsha 410100, China; ^3^College of Mechanical and Electrical Engineering, Central South University, Changsha 410083, China; ^4^Light Alloy Research Institute, Central South University, Changsha 410083, China

## Abstract

We offer a neural network-based control method to control the vibration of the engineering mechanical arm and the trajectory in order to solve the problem of large errors in tracking the path when the engineering mechanical arm is unstable and under the influence of the outside world. A mechanical arm network is used to perform tasks related to learning the unknown dynamic properties of a engineering mechanical arms keyboard without the need for prior learning. Given the dynamic equations of the engineering mechanical arm, the dynamic properties of the mechanical arm were studied using a positive feedback network. The adaptive neural network management system was developed, and the stability and integrity of the closed-loop system were proved by Lyapunov's function. Engineering mechanical arm motion trajectory control errors were modeled and validated in the Matlab/Simulink environment. The simulation results show that the management of the adaptive neural network is able to better control the desired path of the engineering mechanical arm in the presence of external interference, and the fluctuation range of input torque is small. The PID control has a large error in the expected trajectory tracking of the engineering mechanical arm, the fluctuation range of the input torque is as high as 20, and the jitter phenomenon is more serious. The use of detailed comparisons and adaptive neural network monitoring can perform well in manipulating the trajectory of the engineering mechanical arm. The engineering mechanical arm uses an adaptive neural network control method, in which the control precision of engineering mechanical arm motion trajectory can be improved and the out-of-control phenomenon of mechanical arm motion can be reduced.

## 1. Introduction

With the development of science and technology, machine technology is more and more applied in various fields of national life, which puts forward higher requirements for the dynamic performance of machine control system. Machine modeling and control is the main content of machine research and plays a decisive role in the development and application of machines. The machine control object has one or more multi-joint mechanical arms. Its static and dynamic models are extremely complex, with multiple inputs and outputs, which are combined with each other. The system is also closely related to its kinematics and dynamics and is highly nonlinear [1]. During the operation of the machine control system, the system load changes greatly with time and has strong time variability. This kind of complex control system has high requirements on the model of the control object. How to improve the accuracy of modeling is the focus of current research. Many emerging modeling strategies are introduced, which provide a good foundation for improving control performance. Modern industry and engineering construction have higher and higher requirements for mechanical arms, which are reflected in the working speed and precision of the machine [2]. Its appearance makes it inevitable to apply more advanced modern control theory to the field of machine control to solve the problem of nonlinear severe disaster control. Typical advanced modern control theories and technologies include rational control, adaptive control, neural network control, sliding mode variable structure control, and so on. Track control plays an important role in mechanical arm traffic control. In the actual use of the mechanical arm, the system must not only have perfect functions but also have good performance. The first means that the machine must work within a relatively wide range, and the last means that the machine must meet human needs. As a typical representative of engineering machines, mechanical arms have the kinematics and dynamics of general machines, that is, multi-variable, time-varying, random, and extremely nonlinear. The existence of uncertain factors significantly affects the performance of the mechanical arm control system, which is inevitable. Traditional object-based control methods are increasingly difficult to meet the control requirements of mechanical arm trajectory [3, 4].

The development of intelligent management theory makes neural network technology an effective way to solve optimal problems. The reason why the neural network can solve the strong nonlinear mapping problem is determined by its self-organization and self-learning ability. Neural networks can approximate any nonlinear function and have good adaptability to data mapping, which cannot be satisfied by traditional numerical calculation methods [5]. Neural networks are widely used in various areas of automatic control, especially system recognition, nonlinear system control, fault diagnosis, and fault tolerance monitoring, and play a very important role. Nerve networks play a strong role in the recognition of nonlinear systems. As a model for recognizing nonlinear systems, it provides a general model for recognizing neural networks. Therefore, it is the physical realization of the actual system and is suitable for online control. RBFNN based on RBF (radial basis function) is a typical transmission network. It is based on function approximation theory. Network learning is basically to find the optimal plane for data alignment to meet the requirements of multi-dimensional information training space. Any basis function contained in this best-fit plane consists of the latent layer neuron transfer function of the RBFNN. The complexity of mechanical arm kinematics and dynamics makes neural networks very useful and highly applicable in the field of mechanical arm control. The application of neural network technology in mechanical arm control can solve the problem of real-time online control of mechanical arms. On the one hand, by learning the sample data during the actual operation of the mechanical arm, the neural network can obtain the nonlinear relationship between the kinematics or dynamics input and output of the mechanical arm, which is usually represented by a nonlinear expression. The obtained nonlinear relationship can make the system immune to the influence of parameter uncertainty on the control because its establishment process does not directly depend on the actual parameters of the mechanical arm. On the other hand, the neural network control system is robust, which reduces its sensitivity to the system characteristics or parameter distortion of the mechanical arm, making the mechanical arm more intelligent, so as to achieve the purpose of real-time control of the mechanical arm. Neural network is a relatively systematic and accurate control method in the trajectory tracking control of mechanical arm, which provides an effective way to study the modeling and control of mechanical arm. It can ensure that the position and velocity tracking errors of the end effector of the mechanical arm gradually converge to zero [6, 7]. Aiming at the problems of closed-chain structure, unknown dynamic parameters, and strong coupling in coordinated multi-mechanical arm systems, a position-force hybrid control strategy was designed based on fuzzy neural network. The whole system is modeled dynamically in the task space, and the idea of position-force hybrid control is adopted. The controllers are designed in the position space and the force space, respectively. By introducing the coordinated control amount into the position controller, the engineering mechanical arm is reduced in size.

## 2. Related Works

Neural network originated in the early 1940s and has experienced nearly 20 years of development. It was not until the late 1950s that the neural network theory was initially formed. For the problem of mechanical arm modeling and control, many researchers have done a lot of research and successfully verified it in experiments or practice. Schwartz-Leyzac and others proposed an online identification method of mechanical arm dynamic model based on RBFNN and achieved good results on 6-DOF industrial mechanical arm [8]. RBF and MLP (multi-layer perceptron) have been successfully used in 6-DOF series mechanical arms, offering a mechanical armic reverse kinematic solution based on a combination of neural networks [9]. Carney and others studied a new method of adaptive control of neural networks that has been studied and successfully used to control the movement of the PUMA560 mechanical arm [10]. Hsu and others offered a hybrid learning algorithm that combines neural network-based Kalman filters and BP algorithms, combined neural networks with traditional PID control used in mechanical armic adaptive control systems, and explored effective heuristic learning algorithms [11]. Abbas and others made efforts in the study of manipulative neural network management, and a model of adaptive neural network management of mechanical arms based on dynamic return networks was proposed [12]. A stable mechanical armic neural network controller was studied, and a control circuit consisting of a neural network management and control controller was proposed [13]. Patocka and others put forward an adaptive neural slip mode control method to track the path of uncertain mechanical arms, which effectively performs rapid control of the mechanical arm's path under system uncertainty [14]. Liu and others proposed a kinematic model of a mechanical arm that effectively solves the problem of mechanical arm control by offering a dynamic repetitive neural network with state delay input [15]. Blacker and others proposed a solution method of forward and inverse kinematics of mechanical arm based on RBFNN from the perspective of geometry and mathematics, which effectively avoided the tediousness of traditional mechanical arm kinematics solution methods and provided convenience for the research of mechanical arm dynamics control [16]. Fu and others recommended RBFNN-based iteration training controller, which effectively solves the problem of low wristband speed when using iterative training control to control the mechanical arm track [17]. Liu and others put forward a hybrid controller consisting of an adaptive RBFNN and a PD controller, which effectively solves the powerful control problem of controlling the path of an unknown mechanical arm in the event of an external failure [18].

Based on this research, the invention provides a vibration and trajectory control engineering manipulator based on neural network. Starting from the kinematics and dynamics of engineering mechanical arm, this paper studies the RBFNN learning algorithm based on EC-RBF, applies it to the mechanical arm motion control algorithm, realizes the neural network solution of the inverse kinematics of mechanical arm, and designs the mechanical arm NNMARC based on the learning algorithm. The neural network learning method combining offline learning and online adjustment is used to identify the dynamic model of the mechanical arm. Finally, the control of the engineering mechanical arm track is realized.

## 3. Research Methods

### 3.1. Dynamic Characteristics of Engineering Mechanical Arm

The engineering mechanical arm is a multi-link mechanism with an open chain, the main body is fixed on the construction machinery, and an actuator is installed at the free end to suit the needs of the work. The two joints are connected by a rotating joint or a moving joint, and the interaction of the joints involves the movement of the joint and the movement of the mechanical arm to reach a different position. The kinematic equation of the engineering mechanical arm is the conversion of the mechanical arm end effect between the joint space and the working space. The dynamic equation represents the nonlinear relationship between the torque applied to each joint of the engineering mechanical arm and the angular displacement and angular velocity of each joint, that is, the movement of the engineering mechanical arm is controlled by controlling the torque. In order to achieve the goal of real-time monitoring, it is necessary to create a dynamic model of the mechanical arm and effectively monitor it. The main content of the study of the kinematics of the engineering mechanical arm is the creation of a coordinate system of the coordinates of the position of each link, the direction, and the relationship between the transitions between the different coordinates. The kinematics of a engineering mechanical arm has two problems, one is the forward kinematic problem and the other is the reverse kinematic problem, called reverse kinematics. The forward kinematics of the mechanical arm depends on the spatial position and orientation of the mechanical arm, taking into account each of the joint variables of the engineering mechanical arm. Reverse kinematics is the solution of each joint variable according to the spatial position and orientation of the engineering mechanical arm. Both the front kinematic policy of the mechanical arm and the problem of reverse kinematics can be considered as problems of nonlinear mapping of the engineering mechanical arm between the joint distance and the work area [19].

For the engineering mechanical arm with n degrees of freedom, the connection position variable is represented by *q*=[*q*_1_, *q*_2_,…, *q*_*n*_]^*T*^. It is assumed that the research object can be represented by *m* variables *x*=[*x*_1_, *x*_2_,…, *x*_*m*_]^*T*^(*m* < *n*), and *x* = *f* (*q*), where *f* is the forward kinematics equation. After the quadratic differentiation of *X*, we can get

(1) $$\begin{array}{c} x^{\prime \prime }=J^{\prime }\left(q\right)q^{\prime }+J\left(q\right)q^{\prime \prime }, \end{array}$$

where *J*(*q*)=*∂f*(*q*)/*∂q* and *J*(*q*) ∈ *R*^*m*×*n*^ represents the Jacobian matrix of the mechanical arm.

The pseudo-inverse matrix (represented by $\overset{+}{J}\left(q\right)$) of the Jacobian matrix (JQ) of the mechanical arm is defined as follows:

(2) $$\begin{array}{c} \overset{+}{J}=\overset{T}{J}{\left(J\overset{T}{J}\right)}^{-1}, \end{array}$$

where $J\overset{+}{J}=I_{m}$ and *I*_*m*_ ∈ *R*^*m*×*n*^ is the identity matrix.

For the engineering mechanical arm with n connecting pairs, the rotation direct drive can be expressed by the following relationship:

(3) $$\begin{array}{c} M\left(q\right)q^{\prime \prime }+V_{m}\left(q,q^{\prime }\right)+G\left(q\right)+F\left(q^{\prime }\right)=\tau , \end{array}$$

where *M*(*q*) ∈ *R*^*n*×*n*^ is the inertia matrix; *V*_*m*_(*q*, *q*′) is the centripetal Coriolis force matrix; *G*(*q*) ∈ *R*^*n*^ is the gravity matrix; *E*(*q*′) is the friction matrix; and *τ* ∈ *R*^*n*^ is the torque input vector.

Engineering mechanical arm dynamics refer to the study of the relationship between the driving force of each joint and the motion of the joint. The engineering mechanical arm is composed of multiple joints and multiple connecting rods.

Each degree of freedom is driven by a separate actuator. From the control point of view, the engineering mechanical arm control system itself is an automatic control system, which has the characteristics of multi-input and multi-output, nonlinearity, redundancy, and combination. The combination of dynamics is reflected in the relationship between the joint motion of the engineering mechanical arm and the torque applied to each joint. Therefore, the motion control task of the engineering mechanical arm is essentially a dynamic task. There is a strong combination between them, and the parameters are time-varying, and the model is also uncertain. The purpose of studying the dynamics of engineering mechanical arm is to control. In order to realize the accurate control of the motion of the mechanical arm, its dynamic model must be established. Dynamic model identification is one of the most basic but relatively difficult problems in the research of engineering mechanical arm control. There are two main methods to analyze the dynamic model of engineering mechanical arm.

#### 3.1.1. Newton–Euler Equation

This method is based on the dynamic balance method of force, which can eliminate the internal force between joints while obtaining the motion speed of the mechanical arm. This method can be used to analyze the general simple engineering mechanical arm system. For more complex systems, this method will appear extremely complex, so it is not applicable.

#### 3.1.2. Lagrange Equation

It is the main equation of Lagrangian mechanics, which can be used to describe the motion of objects, especially for the study of theoretical physics. The Lagrange equation is functionally equivalent to Newton's second law in Newtonian mechanics. This method is based on Lagrange function balance method. In the establishment process, it is not necessary to obtain the internal force between the joints of the mechanical arm. The derivation is simple and clear, and the physical meaning of the parameters is clear. It is a method with strong applicability. This section will focus on the establishment of the dynamic model of the engineering mechanical arm based on this method.

### 3.2. Feedforward Neural Network

**Figure 1:**
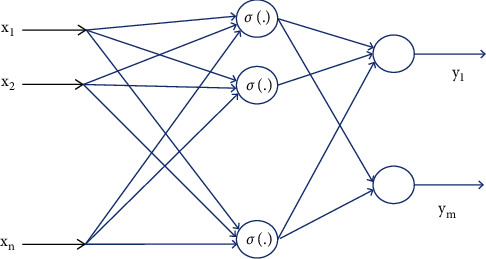
Feedforward neural network.

The neural network is the simplest type of neural network. Each neuron is located in a layer, and each neuron communicates only with neurons in the previous layer. The output of the previous layer is received and passed to the next layer without feedback between the layers. It is one of the most widely used and fastest growing neural networks. Research began in the 1960s and reached a very high level of theoretical research and practical application. The feedforward neural network (FNN) is a type of artificial neural network called the feeder network. The transmission neural network uses a unilateral multi-layer structure. Each layer contains several neurons. In this type of neural network, each neuron can receive signals from nerve cells in the previous layer and output them to the next layer. Layer 0 is called the input layer, the last layer is called the output layer, and the other intermediate layer is called the hidden layer. The hidden layer can be a single layer. It can also be a multi-layer conductive neural network with n input units, m output units, and *N* latent level units, as shown in Figure 1.

The output vector *Z* can be determined by the input vector *X* through the following formula:

(4) $$\begin{array}{c} z_{i}=\sum_{j=1}^{N}\left[\omega_{ij}\sigma \left(\sum_{k=1}^{n}\upsilon_{jk}y_{k}+\theta_{vj}\right)+\theta_{\omega i}\right],\;i=1,2,\text{…},m, \end{array}$$

where *σ*(.) is the hidden layer neuron activation function, *σ*(*y*) = 1/(1 + *e*^−*y*^); *υ*_*jk*_ is the input layer to hidden layer interconnection weight; *ω*_*ij*_ is the weight of the connection between the hidden layer and the output layer; and *θ*_*vj*_, *θ*_*ωi*_ represent deviation weight.

By substituting the neural network weight values *υ*_*jk*_ and *ω*_*ij*_ into the weight matrices *V*^*T*^ and *W*^*T*^, respectively, the corresponding vectors of the neural network equation can be obtained as follows:

(5) $$\begin{array}{c} z=W^{T}\sigma \left(V^{T}y\right), \end{array}$$

where *σ*(*x*)=[*σ*(*x*_1_), *σ*(*x*_2_),…, *σ*(*x*_*n*_)]^*T*^ is the activation function vector, *x* ∈ *R*^*n*^.

The deviation weight value is used as the first column parameter of the weight matrix. In order to accommodate the deviation weight value, the vectors *y* and *σ*(.) need to be expanded accordingly.

**Property 1 .**

Set the smoothing function from *R*^*n*^ to *R*^*m*^ to be h(y) and *U*_*y*_⊆*R*^*n*^. Then, under the condition of *y* ∈ *U*_*y*_ and *ε* > 0, there are *N* hidden layer neurons and weight matrices *W* and *V*, and the relationship is *h*(*y*)=*W*^*T*^*σ*(*VTy*)+*ε*. Then, the estimation of the smoothing function *h* (*y*) can be expressed as $\hat{h}\left(y\right)={\hat{W}}^{T}\sigma \left({\hat{V}}^{T}y\right)$, and $\hat{W}$ and $\hat{V}$ are the estimation matrices of ideal neuron weights obtained by the online weight adjustment algorithm.

### 3.3. Engineering Mechanical Arm Neural Network Control

#### 3.3.1. Neural Network Control

**Figure 2:**
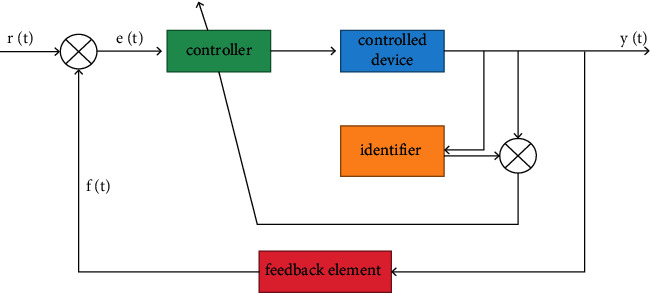
General structure of neural network control system *R* (*T*).

NNC (neural network control), also known as artificial neural network control system, neural network-based control, or neural control for short, refers to the use of neural network to realize the modeling, control, and optimization calculation of complex nonlinear objects. Such a control system is called neural network control system. The main system form of neural network control system is negative feedback regulation. The basic structure of the system is divided into open loop and closed loop. Its general structure is shown in Figure 2. The controller, identifier, and feedback link in the figure can be composed of neural network. Neural network uses binary logic, which is a digital network. Neural control is digital control, which uses digital quantity to control the controlled object. The neural network control system is a kind of digital control system, which has the general characteristics of the digital control system. Like the general microcomputer control system, it also has two parts: hardware and software. Neural network control has fundamentally changed the design idea of the traditional control system and has become a control method without mathematical model of controlled image [20].

In the control system, the neural network functions either as a controller or as an identifier. Neural controller has intelligent performance, so the neural network control system, as an intelligent control system, is a system with learning ability, also known as learning control system. The learning process is a process of training and memorizing the training results. Compared with classical controller and modern controller, neural controller has obvious advantages and disadvantages. The biggest advantage is that the design of neural controller has nothing to do with the mathematical model of the controlled object, which is the fundamental reason why neural network can stand in automatic control. The disadvantage is that the neural network needs to carry out learning and training online or offline and use the training results to design the system. This kind of training largely depends on the accuracy of training samples, and the selection of training samples still has human factors. Neural identifier is used to identify the nonlinearity and uncertainty of the controlled object.

#### 3.3.2. Neural Network Control Method of Engineering Mechanical Arm

**Figure 3:**
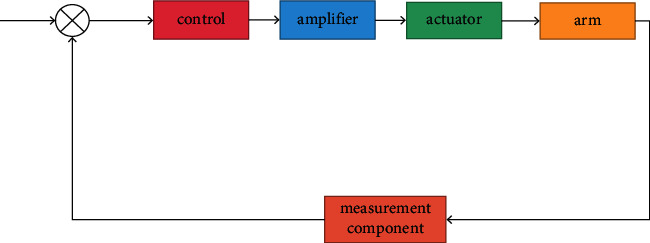
Control structure of mechanical arm.

The basic control of the mechanical arm includes position control, torque control, and force/position hybrid control. Most engineering mechanical arms adopt the control structure based on joint space. Firstly, the joint variables are obtained through inverse kinematics, and then the servo control is realized with the expectation of joint displacement, velocity, and acceleration. The control structure is shown in Figure 3.

In this paper, the research of engineering mechanical arm control starts from position control. Position control mainly refers to the method of the control system when the engineering mechanical arm tracks and controls the trajectory. These include control systems based mainly on switching variables and position-based control systems. The former is studied in joint space and the latter is in operation space. Among them, the control system with shutdown variable is widely used.

**Figure 4:**
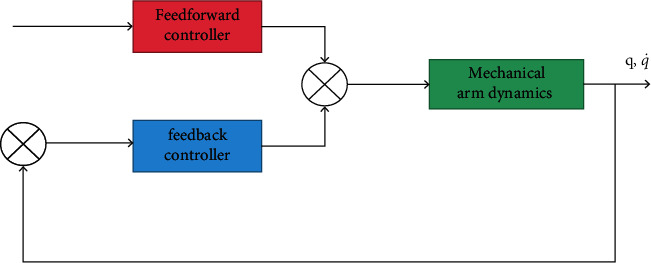
Dynamic control structure of mechanical arm.

In the control system based on joint variables, *q*_*d*_=[*q*_*d*1_, *q*_*d*2_,…, *q*_*dn*_] is the expected joint position vector, ${\dot{q}}_{d}$ and ${\ddot{q}}_{d}$ are the expected joint velocity and acceleration, *u*_1_ and *u*_2_ are control quantities, and *f*=[*f*_1_, *f*_2_,…, *f*_*n*_] is the driving torque vector of the joint. The function of the control system is to find the appropriate control quantity to determine the driving torque required by the driving joint, so as to achieve the purpose of tracking the desired trajectory [21]. The above control process is based on the dynamics of the mechanical arm, so the control problem turns to the dynamics control of the mechanical arm. The traditional dynamics control structure is shown in Figure 4.

**Figure 5:**
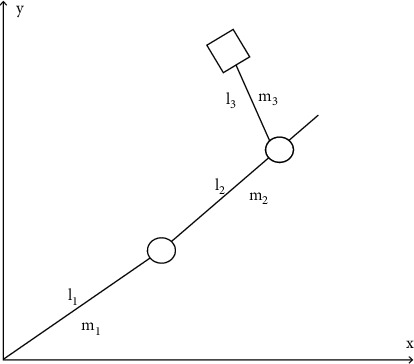
Schematic diagram of mechanical arm model.

In Figure 5, *q*, $\dot{q}$ represent the joint position and speed of the engineering mechanical arm. As can be seen from the figure, the engineering mechanical arm controller includes a transmitter controller and a feedback controller, the most important part of which is the design of the transmitter controller. How the controller is designed to support the operation of the control system depends largely on the dynamic design of the engineering mechanical arm. If the model is clearly known, the traditional methods such as moment calculation method and accurate linearization method can meet the requirements. However, when the model is unknown, the control system actually becomes the traditional PID control. Obviously, such a control system is difficult to obtain good control performance. Using certain methods to eliminate or reduce the influence of the uncertainty of the engineering mechanical arm dynamic model on the control system, so as to improve the performance of the control system, has become a problem that researchers need to solve. Thus, the adaptive method is effectively applied to it. Adaptive control can effectively overcome the structural uncertainty, compensate the influence of nonlinear and uncertain factors on the system, and can significantly improve the performance of the engineering mechanical arm. However, adaptive control is not effective for nonstructural engineering manipulator. In order to solve this problem, researchers have made a lot of exploration and opened up a new idea of control with the help of neural network. Neural network can become a powerful tool for engineering mechanical arm control, which is determined by its strong nonlinear mapping ability, self-learning ability, and adaptive ability [22, 23].

## 4. Result Discussion

The established schematic diagram of the engineering mechanical arm model is simulated and verified, as shown in Figure 5.

**Figure 6:**
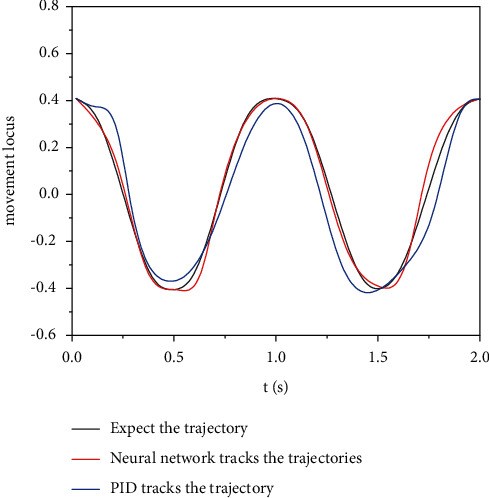
Movement track of engineering mechanical arm 3 (without external interference).

**Figure 7:**
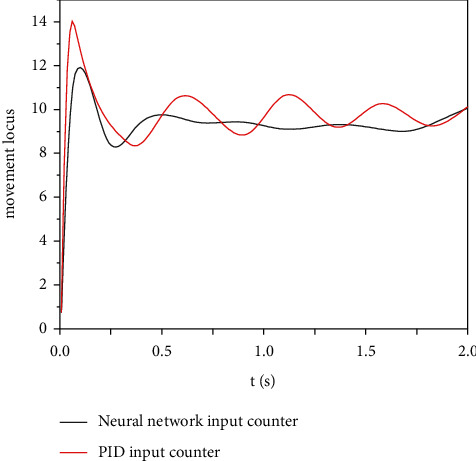
Torque control of engineering mechanical arm 3 (no external interference).

**Figure 8:**
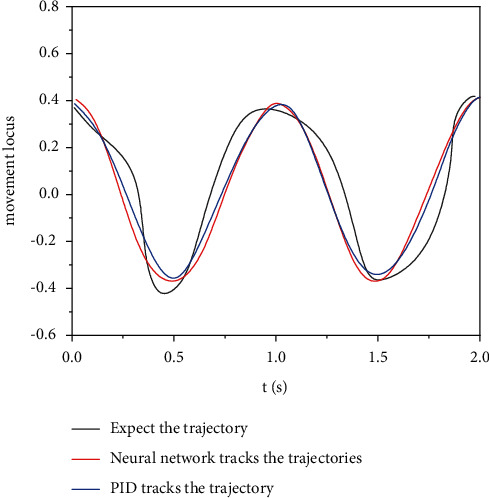
Movement track of engineering mechanical arm 3 (with external interference).

**Figure 9:**
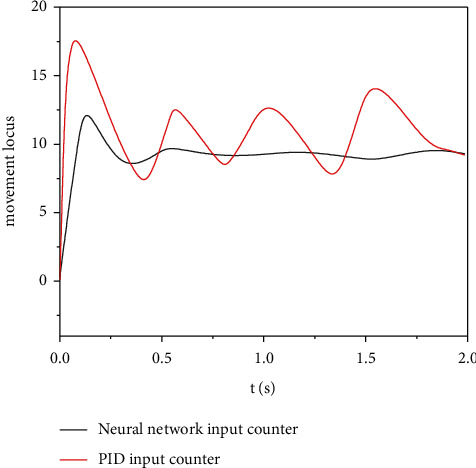
Torque control of engineering mechanical arm 3 (with external interference).

The tracking error of the engineering mechanical arm movement trajectory was modeled in the Matlab/Simulink environment to test the tracking effect of the mechanical arm motion trajectory controlled by the adaptive neural network. The simulation results of motion track tracking error of engineering mechanical arm 3 are obtained. The simulation results of mechanical arm 1 and mechanical arm 2 are similar to those of mechanical arm 3, so simulation verification is not performed here. The simulation parameters are set as follows: the expected trajectory of mechanical arm 3 is *θ*_3_ = 0.4  cos(2*πt*), the initial condition is *θ*(0) = [000]^*T*^, the control parameter *k* = diag (40, 40, 40), the interference parameter *τ*′ = 20  cos(*πt*), and the mechanical arm link parameters are*L*1 = 0.62 M, *L*2 = 0.41 m, *L*3 = 0.34 M, *M*1 = 3.5, *M*2 = 2.5 kg, *m*3 = 2.0 kg, *g* = 9.82 m/s^2^, and *t* = 2 S. Under the condition of no external interference, the simulation results of motion trajectory tracking of engineering mechanical arm 3 are shown in Figure 6, and the simulation results of input torque of engineering mechanical arm 3 are shown in Figure 7. In the case of external interference, the trajectory tracking simulation results of engineering mechanical arm 3 are shown in Figure 8. The simulation results of input torque of engineering mechanical arm 3 are shown in Figure 9.

It can be seen from Figures 6 and 7 that under the condition of no external interference, both adaptive neural network control and PID control can effectively track the desired trajectory of the engineering mechanical arm. Using PID control, the input torque fluctuates greatly and the jitter phenomenon is serious. Therefore, the adaptive neural network control method is better. It can be seen from Figures 8 and 9 that under the condition of external interference, the adaptive neural network control can better track the desired trajectory of the engineering mechanical arm, and the fluctuation range of input torque is small. However, the PID control has a very large tracking error in the expected trajectory path of the engineering mechanical arm, the range of input torque fluctuations is up to 20, and the jitter phenomenon is more serious. Adaptive neural network monitoring can be used to control the trajectory of engineering manipulator.

## 5. Conclusion

The modeling and control of a nonlinear dynamic system based on neural network is a frontier subject of nonlinear discipline, which is of great significance in many fields, especially for the multi-variable and strong disaster uncertain nonlinear object such as mechanical arm. This article examines the control of the neural network to control the trajectory of the engineering mechanical arm. The neural network controller can effectively control the pathway of the end effector and control the subtask of the program. Given the dynamic equations of the engineering mechanical arm, the dynamic properties of the engineering mechanical arm were studied using a positive feedback network. The adaptive neural network management system was developed, and the stability and integrity of the closed-loop system were proved by Lyapunov's function. We created a schematic diagram of the engineering mechanical arm model and simulated the dynamic parameters of the engineering mechanical arm using the Matlab/Simulink program. In addition, the PID control system simulation results are compared and analyzed. The simulation results show that in the case of external interference, the adaptive neural network control method can not only accurately realize the trajectory tracking task of the mechanical arm but also effectively weaken the jitter of the engineering mechanical arm.

## Data Availability

The data used to support the findings of this study are available from the corresponding author upon request.
